# Bullae and pigmentary skin changes revealing AL amyloidosis: A case report

**DOI:** 10.1016/j.jdcr.2026.06.028

**Published:** 2026-06-18

**Authors:** Sara Mubarak, Danah I. AlKandari, Hayam AlEnezi, Al-Sadat Mosbeh, Mohammad J. AlKandari, Fatmah AlHendi

**Affiliations:** a Department of Dermatology, Adan Hospital, Al Ahmadi, Kuwait; b Department of Dermatology, Jaber Al Ahmad Hospital, Mubarak Al Kabeer, Kuwait; c Professor of Dermatology & Dermatopathology, Al-Azhar University, Cairo, Egypt; d Department of Pathology, Farwaniya Hospital, Al Farwaniya, Kuwait; e Department of Oral Medicine, Farwaniya Specialized Dental Center, Al Farwaniya, Kuwait

**Keywords:** bullous amyloidosis, cutaneous amyloidosis, dyschromia, immunoglobulin light chain (AL) amyloidosis, macroglossia

## Introduction

Amyloidosis refers to a group of disorders characterized by extracellular deposition of fibrillar proteins that may result in organ dysfunction. Amyloidosis is classified clinically according to the number of involved organs into localized and systemic forms.^1^^,^^2^ Localized amyloidosis is confined to a single organ, such as the skin, resulting in macular, lichen, nodular, or dyschromic forms.^3^ Systemic amyloidosis involves multiple organs and is subclassified according to the precursor protein into amyloidosis (AL), amyloid A amyloidosis, and transthyretin amyloidosis amyloidosis. AL amyloidosis can be idiopathic or associated with plasma cell dyscrasias. In contrast, AA amyloidosis or secondary systemic amyloidosis is caused by increased serum amyloid A production in the setting of chronic infection or inflammation. Transthyretin amyloidosis amyloidosis results from transthyretin protein deposition and is divided into wild-type and hereditary forms.^1^^,^^2^

Common mucocutaneous manifestations of AL amyloidosis include macroglossia, purpura, and ecchymoses.^1^^,^^4^, ^5^, ^6^ We present a case of AL amyloidosis with unusual cutaneous manifestations, including bullous lesions and dyschromia. To our knowledge, dyschromia has not been previously reported in association with AL amyloidosis.

## Case report

A 68-year-old woman presented to the dermatology department with a 1-year history of asymptomatic blisters on the lower extremities. Her medical history was significant for type 2 diabetes mellitus, hypothyroidism, hypertension, and dyslipidemia. The patient’s medications included metformin, amlodipine, telmisartan/hydrochlorothiazide, atorvastatin, levothyroxine, and allopurinol. Physical examination revealed tense bullae on a background of mottled hyperpigmented and hypopigmented patches and macules, hemorrhagic crusts, and excoriations on both legs (Fig 1). Concurrently, she reported a 4-month history of fatigue, hoarseness of voice, and an enlarged tongue causing difficulty breathing and eating. Oral examination revealed macroglossia with a rubbery scalloped tongue (Fig 1). The initial differential diagnoses included bullous pemphigoid, edema blisters, and diabetic bullae. The differential diagnosis for the macroglossia included drug-induced macroglossia, acromegaly, and systemic amyloidosis.

**Fig 1:**
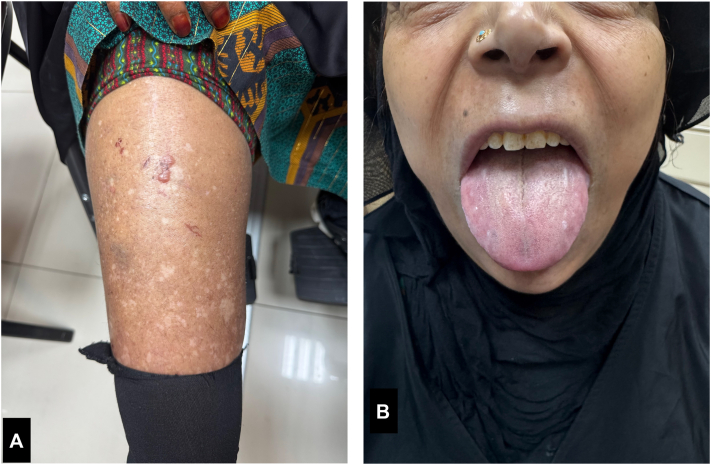
Hyperpigmented macules, hypopigmented macules and patches with a tense hemorrhagic blister, crusts and excoriations on the right shin **(A)**. Macroglossia with tooth indentations along the lateral border of the tongue **(B)**.

Punch biopsies were obtained from the edge of the blister, a hyperpigmented macule, and a hypopigmented macule. Histopathologic examination of the blister biopsy showed intraepidermal and subepidermal clefts with superficial perivascular inflammatory infiltrate (Fig 2). Examination of the hyperpigmented macule revealed epidermal atrophy, increased basal layer melanin deposition, and perivascular lymphocytic infiltrates (Fig 3). The hypopigmented macule showed hyperkeratosis with epidermal atrophy and decreased basal layer melanin pigmentation. Congo red staining of the biopsies obtained from the blister and hyperpigmented macule demonstrated amyloid deposition in the papillary dermis (Figs 2 and 3). Enzyme-linked immunosorbent assay, direct immunofluorescence, and indirect immunofluorescence studies were negative for autoimmune bullous diseases. Tongue biopsy demonstrated amyloid deposits within the lamina propria, skeletal muscle, and around small blood vessels. Congo red staining demonstrated characteristic green birefringence under polarized light microscopy (Fig 4).

**Fig 2:**
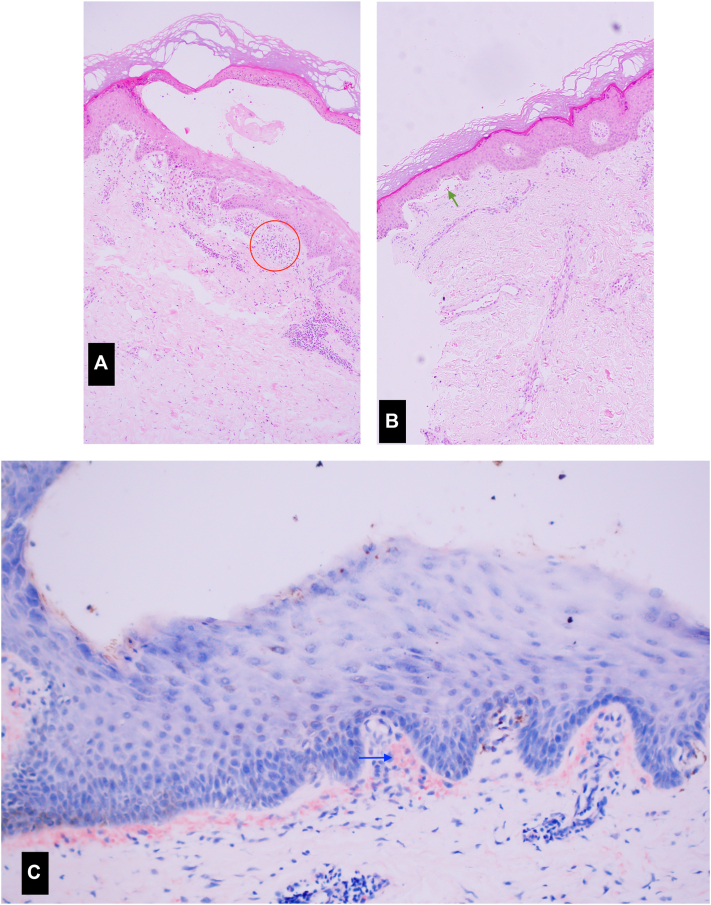
Blister biopsy revealed intraepidermal blister with superficial perivascular inflammatory infiltrate composed of lymphohistiocytes with melanophages (*red circle*) (**A,** H&E, 10×). Subepidermal blister containing RBCs (*green arrow*) (**B,** H&E, 10×). Orange-red staining of amyloid deposits in papillary dermis (*blue arrow*), (**C,** Congo red stain, 10×).

**Fig 3:**
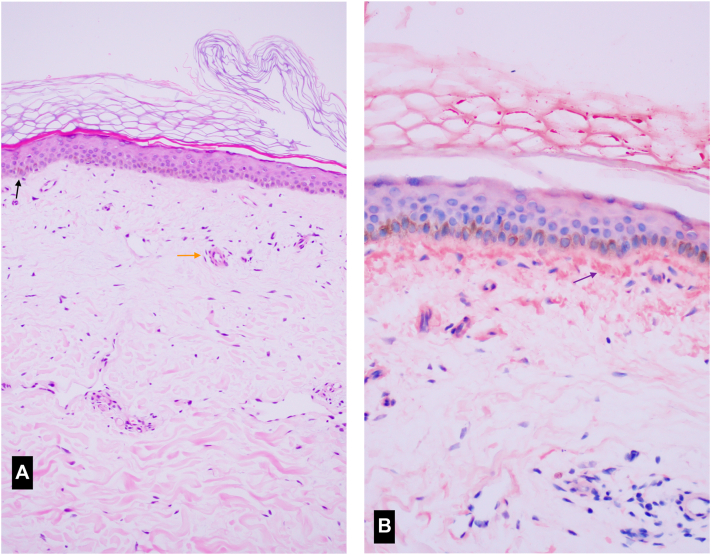
Biopsy from a hyperpigmented macule. **A,** Epidermal atrophy, increased melanin deposition within the basal layer (*black arrow*), and sparse superficial perivascular lymphohistiocytic inflammatory infiltrate (*orange arrow*) (H&E, 10×). **B,** Increased basal pigmentation and amyloid deposits in papillary dermis (*purple arrow*) (Congo red stain, 20×).

**Fig 4:**
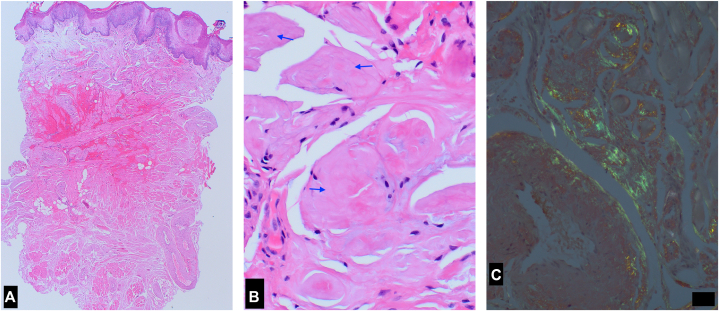
Tongue biopsy demonstrated extracellular deposition of amorphous, eosinophilic, acellular material within the lamina propria, skeletal muscle, and around small blood vessels (**A,***blue circles*) (**B,***blue arrows*). Congo red stain demonstrated apple-green birefringence under polarized light microscopy **(C)**.

Laboratory investigations, including complete blood count, liver and renal function panels, and thyroid function tests were normal. Hepatitis screening and QuantiFERON-TB Gold testing were nonreactive. Free light chain analysis revealed a lambda (λ) level of 784 mg/L (normal range, 8.3-27 mg/L) and a kappa (κ) level of 11 mg/L (normal range, 6.7-22.4 mg/L). The serum κ:λ ratio was 0.014 (normal range, 0.3-1.6). The 24-hour urine protein level was 211 mg/24 h (10-125 mg/24 h). Bone marrow aspirate showed a normocellular marrow with trilineage hematopoiesis and 8% plasma cells. Flow cytometry identified plasma cells comprising 2% of total cells. Bone marrow biopsy revealed a plasma cell infiltrate of 35% to 40% with positivity for CD38 and CD56.

Magnetic resonance imaging of the brain and neck revealed a diffusely enlarged tongue with mild narrowing of the oropharyngeal airway at the level of C2. Magnetic resonance imaging of the cervical and dorso-lumbar spine, chest x-ray, skeletal survey, whole-body positron emission tomography/computed tomography scan, and abdominal and thyroid ultrasonography were non-contributory. Transthoracic echocardiography demonstrated grade 2 diastolic dysfunction with mild concentric left ventricular hypertrophy, normal left ventricular ejection fraction, mild left atrial dilatation, and normal strain patterns. Stress myocardial perfusion imaging showed 2 areas of reversible ischemia in the anterolateral wall extending to the apex and anteroseptal wall.

After excluding secondary causes of systemic amyloidosis, the patient was diagnosed with AL amyloidosis presenting with bullous amyloidosis, dyschromia, macroglossia, and echocardiographic findings suggestive of early cardiac involvement. She was referred to the oncology center and treated with daratumumab, cyclophosphamide, bortezomib, and dexamethasone. Six months after treatment initiation, the patient showed mild improvement in macroglossia and no new blisters.

## Discussion

AL amyloidosis is a rare disease caused by the production of insoluble light-chain proteins by plasma cell clones in the bone marrow, which may deposit in any organ except the central nervous system.^2^^,^^4^, ^5^, ^6^ The nonspecific initial symptoms of AL amyloidosis usually lead to diagnostic delay. These symptoms include fatigue, shortness of breath, weight loss, edema, syncope, paresthesias, and orthostatic hypotension. The heart and kidneys are the most frequently involved organs, affecting 76% and 53% of patients, respectively.^2^ The liver and peripheral nerves are less commonly involved.^2^^,^^4^, ^5^, ^6^

Amyloid deposits can occur in the upper aerodigestive tract, most often affecting the tongue and larynx. Tongue involvement can cause hemorrhagic papulonodules or macroglossia with scalloped dental indentations, leading to speech and swallowing difficulties, obstructive sleep apnea, and jaw malocclusion.^1^^,^^4^^,^^6^ Skin involvement occurs in 25% of AL amyloidosis cases. It usually presents as purpura and waxy, skin-colored papules and nodules.^7^, ^8^, ^9^ Cases of alopecia, nail dystrophy, and scleroderma-like changes have been reported in association with AL amyloidosis.^8^^,^^9^ Bullous amyloidosis can be the initial sign of AL amyloidosis, although it is rare. It is usually characterized by tense blisters of variable sizes, which can be hemorrhagic and involve the skin and oral mucosa. The exact mechanism of blister formation is unknown, although bullae may result from subepidermal or intradermal clefts.^7^, ^8^, ^9^

Detection of amyloid deposits in a mucocutaneous biopsy warrants further workup. This includes a detailed systemic review and organ-directed investigations. To rule out plasma cell dyscrasias, serum and urine protein electrophoresis, immunofixation electrophoresis, and free light chain assay should be performed. If monoclonal proteins are present, abdominal fat pad aspiration and bone marrow aspirate and biopsy are recommended. If monoclonal proteins are undetectable but AL amyloidosis is still suspected, a biopsy of the affected organ (eg, liver or kidney) is recommended. The diagnosis is confirmed by demonstrating amyloid deposition in tissue using light microscopy with hematoxylin and eosin and Congo red staining or by electron microscopy.^2^^,^^4^^,^^10^

Treatment of AL amyloidosis is stage-dependent. The standard treatment is a combination of daratumumab, cyclophosphamide, bortezomib, and dexamethasone. Autologous stem cell transplantation can be considered in eligible patients.^5^ AL amyloidosis is associated with poor prognosis, with a median survival of 13 months.^10^

## Conflicts of interest

None disclosed.
